# Leveraging and Harnessing Generative Artificial Intelligence to Mitigate the Burden of Neurodevelopmental Disorders (NDDs) in Children

**DOI:** 10.3390/healthcare13151898

**Published:** 2025-08-04

**Authors:** Obinna Ositadimma Oleribe

**Affiliations:** 1 Department of Health Sciences, School of Public Health and Health Sciences, California State University, Dominguez Hills, Carson, CA 90747, USA; ooleribe@csudh.edu; Tel.: +145-82322750; 2 Center for Family Health Initiative (CFHI), Orange, CA 92865, USA

**Keywords:** Generative Artificial Intelligence (GenAI), neurodevelopmental disorders (NDDs), neurodevelopmental care, early detection, diagnostic precision, personalized treatment, inclusive research, caregiver support, evidence-based interventions

## Abstract

Neurodevelopmental disorders (NDDs) significantly impact children’s health and development. They pose a substantial burden to families and the healthcare system. Challenges in early identification, accurate and timely diagnosis, and effective treatment persist due to overlapping symptoms, lack of appropriate diagnostic biomarkers, significant stigma and discrimination, and systemic barriers. Generative Artificial Intelligence (GenAI) offers promising solutions to these challenges by enhancing screening, diagnosis, personalized treatment, and research. Although GenAI is already in use in some aspects of NDD management, effective and strategic leveraging of evolving AI tools and resources will enhance early identification and screening, reduce diagnostic processing by up to 90%, and improve clinical decision support. Proper use of GenAI will ensure individualized therapy regimens with demonstrated 36% improvement in at least one objective attention measure compared to baseline and 81–84% accuracy relative to clinician-generated plans, customize learning materials, and deliver better treatment monitoring. GenAI will also accelerate NDD-specific research and innovation with significant time savings, as well as provide tailored family support systems. Finally, it will significantly reduce the mortality and morbidity associated with NDDs. This article explores the potential of GenAI in transforming NDD management and calls for policy initiatives to integrate GenAI into NDD management systems.

## 1. Introduction

Neurodevelopmental disorders (NDDs) represent a significant global challenge to child health, with profound implications for developmental, social, educational, and emotional well-being [1,2,3,4,5]. NDDs such as autism spectrum disorder (ASD), attention-deficit/hyperactivity disorder (ADHD), intellectual disability (ID), and specific learning disorders (SLDs), including dyslexia, dysgraphia, and dyscalculia, are highly prevalent and frequently comorbid, often resulting in lifelong challenges for affected individuals and their families [1,2]. Globally, about 1 in 100 children has ASD [6,7], and ADHD is present in 8.0% of children and adolescents, twice as high in boys (10%) compared to girls (5%) [8]. In the United States, about 1 in 6 (17%) children aged 3–17 years have a developmental disability, 1 in 31 (3.2%) aged 8 years have ASD, and an estimated 7 million (11.4%) aged 3–17 years were diagnosed with ADHD in 2022 [3,4]. In Asia, the overall prevalence of ASD is 0.36%, with prevalence higher in East Asia (0.51%) than in West Asia (0.35%) and South Asia (0.31%) [9]. Current figures likely underestimate the true burden, as many children, particularly those from low-resource or culturally diverse backgrounds, remain undiagnosed or misdiagnosed due to persistent challenges in access, awareness, and culturally responsive diagnostic tools [3,4,5], making the prevalence of NDDs in many low- and middle-income countries unknown. A review of the global prevalence of ASD did not identify any data from sub-Saharan Africa (SSA), even though this region has a population of nearly 1 billion, 40% of whom are children younger than 14 years [10,11].

Beyond the more commonly recognized disorders, the neurodevelopmental landscape also includes communication, developmental coordination, and tic disorders such as Tourette’s Syndrome. These conditions contribute further to the complexity of assessment and treatment, requiring specialized support across educational, clinical, and community settings [1]. As children with ASD commonly present with comorbid ADHD or learning disorders, these comorbidities complicate both diagnosis and management, necessitating integrated and multidisciplinary approaches to care [2].

The impact of NDDs extends beyond the individual, as they place substantial emotional, financial, and logistical burdens on families and healthcare systems. Delays in diagnosis and limited access to evidence-based interventions remain persistent global issues, particularly in low- and middle-income countries (LMIC) where mental health infrastructure is underdeveloped [2]. To improve outcomes, early identification and intervention are crucial. However, many children remain undiagnosed or inadequately supported well into their school years [12].

## 2. Challenges with Neurodevelopmental Disorder (NDD) Management

Despite their profound and wide-reaching impact, the effective management of NDDs remains substantially limited due to the overlapping nature of symptoms across diagnostic categories, the absence of reliable and specific biomarkers, significant delays in recognition and intervention, as well as several systemic, diagnostic, and societal barriers [12,13,14,15]. Furthermore, pervasive stigma, discrimination, and structural inequalities, especially among underserved and marginalized populations, continue to obstruct equitable access to care and support services [2,16]. In addition to diagnostic ambiguity, the variability in the age of symptom onset, heterogeneity in presentation, and the influence of environmental, socioeconomic, and cultural factors further complicate accurate diagnosis and comprehensive care. Thus, many children do not receive timely interventions, resulting in long-term developmental and functional consequences [16].

Similarly, the treatment and management of NDDs are significantly hindered by a global shortage of trained and skilled professionals, limited availability of specialized services, and the complexity of designing effective, individualized interventions. Even in settings with adequate resources, experienced clinicians struggle to establish early and accurate diagnoses due to the absence of standardized biomarkers and the variability in presentation across developmental stages [2,16]. Access to specialized care is frequently restricted by geographic disparities, financial constraints, and limited healthcare infrastructure. These barriers disproportionately affect children in LMIC, rural communities, and underserved populations, where child mental health services are either scarce or altogether unavailable [17,18]. The global workforce trained in child and adolescent neurodevelopmental health is insufficient to meet the growing demand, with many regions reporting critical shortages of developmental pediatricians, child psychologists, speech–language pathologists, and occupational therapists [19].

In addition, families of children with NDDs face considerable and often overwhelming challenges in managing the multifaceted demands of care. These challenges include coordinating between multiple healthcare providers, sustaining adherence to long-term and often intensive treatment plans, and confronting persistent societal stigma and discrimination [20,21]. These complexities frequently necessitate engagement with various specialists, creating a fragmented and poorly integrated system that places a disproportionate logistical and emotional burden on families [22]. Moreover, families must often navigate healthcare systems that lack consistent and universal early screening practices, resulting in delayed identification and intervention.

Inadequate early detection, particularly in underserved and culturally diverse populations, further impedes timely access to services and undermines the potential benefits of early intervention [5,23]. Cultural beliefs and practices, language barriers, absence or near absence of providers with a good understanding of the culture of parents and families further impede access, comprehension of care protocols, and use of vital services. These systemic limitations and barriers lead to gaps in the continuum of care, reinforcing disparities and perpetuating poor outcomes.

Several studies have shown that the use of telehealth has the potential to increase the availability of treatment, decrease waiting times for diagnosis, and aid in the monitoring of NDD [24]. In NDD management, following the COVID-19 pandemic that started in 2019, telehealth was introduced to facilitate care [24]. In the Asian-Pacific region, telehealth packages comprised direct and indirect methods of synchronous, asynchronous, and hybrid approaches, and used parent-led intervention strategies [25]. Other solutions introduced to improve access were mobile screening apps and traditional machine-learning diagnostics. Structural, systemic, and language barriers, economic challenges, including lack of insurance coverage, and unreliable internet access, hindered the full realization of the benefits of these initiatives.

## 3. Leveraging Generative Artificial Intelligence (GenAI) for NDDs Care

In light of these challenges, GenAI presents a transformative opportunity to address the longstanding gaps in the care of NDDs as it rapidly reshapes the healthcare landscape [26,27] and can overcome identified key limitations in NDD care [28,29,30,31].

First, GenAI can make NDD diagnosis easier, faster, and more accurate. By leveraging large-scale data synthesis and pattern recognition, GenAI tools can help reduce diagnostic delays resulting from symptom overlap, lack of biomarkers, and clinician shortages [20]. Intelligent algorithms can analyze multimodal inputs such as behavioral observations, clinical notes, and developmental histories to support early and more precise identification of NDDs [29]. For instance, fMRI-based AI (ASD-DiagNet) increases diagnostic accuracy and reduces diagnosis from traditional 6 h/case to 40 min—90% reduction, behavioral models (e.g., RGB-D) automated classification with accuracy nearing 96% while saving clinicians hours, allowing more automated assessments and avoiding lengthy clinician-led behavioral observation sessions, and retinal/ERG-based AI technology reduced assessment time to about 10 min/case from traditional 60 min, enhancing early intervention, and providing relief for parents and caregivers facing long waitlists [32,33,34]. These improvements reduced labor cost as fewer clinician hours are needed, throughput increases (sometimes doubling), with overall cost savings per clinic ranging in the tens of thousands of dollars annually.

GenAI tools synthesize large volumes of structured and unstructured patient data to generate diagnostic hypotheses and decision support outputs, reduce subjectivity, and improve diagnostic accuracy. By standardizing assessment protocols, these tools reduce inter-clinician variability and mitigate biases that often arise due to cultural, linguistic, or socioeconomic differences [28,35]. Furthermore, AI-driven platforms empower non-specialists, such as parents, educators, and primary care providers, by offering structured digital guidance for early symptom recognition and prompting timely referral to specialists [36]. By democratizing access to early screening and diagnostic tools, GenAI will reduce NDD diagnostic delays, increase equity in access to NDD care, and support earlier, more targeted intervention strategies, especially in LMIC, where there are diverse structural and human resource challenges.

Enabling automated, scalable, and objective assessment mechanisms will enhance early detection and diagnosis, critical for improving outcomes in children with NDDs. Using GenAI systems to analyze video and audio data to identify early behavioral markers such as atypical eye gaze, facial expressions, prosody, and speech patterns will help identify early risk for conditions like ASD or speech–language impairments [31,37]. Automating the analysis of behavioral cues through sophisticated video and speech processing algorithms will enable screening at scale with greater consistency than traditional observational methods [38,39]. Digital symptom checkers guided by validated instruments (e.g., M-CHAT, Vanderbilt ADHD scales) embedded within GenAI platforms will guide caregivers and clinicians through structured assessments, while multimodal data integration combining inputs from electronic health records (EHRs), wearable devices, and caregiver reports will allow for the identification of high-risk profiles with greater sensitivity and specificity [28,40].

Second, GenAI can enable the personalization of therapeutic interventions through adaptive systems that learn from individual responses over time, thereby supporting tailored and responsive care pathways and thus, revolutionizing treatment strategies for NDDs. These systems will empower caregivers and clinicians by providing real-time decision support, automated documentation, and virtual coaching, fostering a more continuous and integrated model of care [30]. They will generate personalized therapeutic regimens by analyzing a child’s comprehensive developmental profile, response history, and co-occurring conditions, which enables clinicians to optimize treatment plans, tailoring interventions to the unique cognitive, behavioral, and emotional needs of each child [27,28]. For example, EndeavorRx (game-based ADHD Rx) demonstrated a 36% improvement in at least one objective attention measure compared to baseline, with no pharmacologic side effects among ADHD children, reducing their reliance on multiple therapy sessions [41]. AI-powered personalized applied behavior analysis (ABA) achieved 81–84% accuracy relative to clinician-generated plans, significantly reducing time spent on manual planning and goal setting for children with ASD [42]. AI-augmented ABA could assist clinicians in focusing on making precise data-driven decisions and increasing the quality of individualized interventions for individuals with NDD [43]. As GenAI synthesizes data from multiple sources, including EHRs, caregiver input, and behavioral tracking tools, to inform decisions about medication management, therapy frequency, and modality selection, GenAI supports adaptive treatment protocols that evolve in response to real-time data, improving the accuracy, responsiveness, and efficiency of care delivery [29,40]. AI systems will also monitor treatment outcomes continuously, assess engagement levels, and automatically recommend modifications, ensuring sustained therapeutic alignment with the child’s evolving needs.

Third, GenAI is being piloted in precision medicine, health education, and several other aspects of NDD management and care [44,45,46]. When operationalized to scale, GenAI will refine customized educational resources and interactive digital content such as visual schedules, gamified learning tasks, or social narratives tailored to individual cognitive and communicative profiles; tools that are particularly beneficial to children with ASD, ADHD, or SLDs, where structured, multimodal, and engaging interventions improve attention, retention, and skill acquisition [37,47]. Current evidence reveals that AI reduced administrators’ work time by approximately 50%, reducing the time staff spend on collecting, cleaning, and analyzing data. The use of AI tools enabled providers to more efficiently allocate resources and inform program decisions; improve children’s engagement, comprehension, and accessibility; reduce teacher and aide support time, thereby accelerating learning pace and reducing repetitive interventions. They also helped reduce missteps and review cycles, indirectly saving instructional hours and accelerating skill acquisition [45,48,49,50].

Scalability is a key advantage of GenAI as it allows for widespread deployment of individualized interventions across diverse care settings, including schools, clinics, and homes. This opens new avenues for delivering high-quality, personalized care to populations traditionally underserved by specialist-intensive systems. As the field continues to evolve, the responsible development and implementation of GenAI tools will be essential to ensure their ethical use, transparency, and inclusivity as they continue to play more significant roles at various levels of NDD care [31,37,38,39,40].

Fourth, GenAI will transform research paradigms by enabling large-scale data integration, pattern recognition, and predictive modeling in NDD. By processing vast, heterogeneous datasets including EHRs, neuroimaging, genetic profiles, behavioral observations, and patient-reported outcomes, GenAI offers unprecedented opportunities to accelerate discovery and deepen understanding of NDD etiology, progression, and treatment response [26,51]. Traditional NDD research has often excluded marginalized groups due to language, geographic, cultural, or socioeconomic barriers, leading to limited generalizability of findings [52].

Multiple studies have emphasized the time-saving benefit of AI use in research. Automating routine tasks frees researchers for more complex work, cuts processing time from around 6 h to just 40 min per case. It also enables predictive analyses on sample sizes of hundreds to thousands, significantly reducing manual chart review, data extraction, and coding time; and achieves higher accuracy (AUC > 75%) in identifying risk profiles for ASD and ADHD in datasets of hundreds of individuals. It also enables the reuse of large, centralized datasets, integrates low-cost EEG collection with cloud analytics to support scalable early screening research, and reduces reliance on costly diagnostic tests or genetic sequencing by extracting relevant features from multimodal sources [53,54,55]. AI adoption in behavioral studies will cut data annotation labor by up to half, accelerating both the annotation and interpretation process for developmental patterns [56,57].

GenAI will support the design and implementation of more inclusive research studies by identifying underrepresented populations and ensuring diverse sociodemographic representation in data sampling and analysis. AI-powered approaches will mitigate (or lower) biases by accounting for variations in presentation across populations and adapting models accordingly, thereby improving the equity and applicability of scientific evidence [35,58]. Also, GenAI will identify novel correlations and latent variables within large datasets, revealing new avenues for hypothesis generation and targeted interventions. This may facilitate early identification of biomarkers, uncover previously unrecognized subtypes, and guide precision medicine strategies tailored to individual and population-level needs [28,40]. By embedding inclusivity into data science methodologies, GenAI not only enhances scientific rigor but also ensures that advances in diagnosis, treatment, and policy formulation are accessible and relevant to all communities impacted by NDDs.

Finally, GenAI will empower families of children with NDDs by facilitating access to timely, comprehensible, and culturally appropriate information. Currently, the diagnosis of ASD can take 12–30 months from first concern to confirmed diagnosis. However, AI-powered tools have reduced the diagnosis time by 30–50% in some pilot studies [59,60]. Studies have shown that AI-driven apps providing behavior management strategies or developmental tracking reduce parental reliance on therapists by 15–25% [61]. AI in pediatric care will contribute billions in annual U.S. healthcare savings as AI can reduce the time parents spend coordinating care by several hours per week, equivalent to thousands of dollars in indirect labor time saved. Other impactful applications of GenAI are the development of accessible, multilingual educational materials that inform caregivers about NDDs, available interventions, and strategies for home-based support [30,58]. These resources are critical for mitigating the informational and emotional burdens commonly experienced by families navigating complex care systems. AI-powered platforms, including conversational agents (chatbots), interactive coaching systems, and mobile health applications, can provide real-time, personalized support to caregivers. These tools will offer continuous guidance, such as answering questions, reinforcing therapeutic strategies, and tracking progress, thereby extending the reach of clinical support beyond traditional settings [36,62]. When available 24/7, these systems will promote treatment adherence, enhance parental confidence, and deliver psychoeducation, which are core components in reducing caregiver stress and burnout [63].

These technologies will strengthen family resilience by fostering shared decision-making. It will also improve communication between caregivers and professionals and increase engagement with therapeutic services. By commonizing and decentralizing knowledge and support, GenAI will democratize care, especially for families in linguistically diverse, rural, or underserved communities [64]. Integrating GenAI into family-centered models of care will offer a scalable and sustainable strategy for promoting caregiver empowerment, enhancing intervention fidelity, and ultimately improving outcomes for children with NDDs.

## 4. Policy Frameworks for GenAI Integration in NDD Care

To fully realize the transformative potential of GenAI in reducing the burden of NDDs, the establishment of comprehensive policy directives and robust ethical frameworks is essential. They must support existing frameworks as well as address the unique challenges and sensitivities inherent in pediatric neurodevelopmental care while facilitating the responsible adoption of AI technologies in clinical practice [65,66,67,68,69].

In October 2023, the World Health Organization (WHO) released comprehensive guidance to support member states’ responsible regulation of AI technologies in healthcare. This guidance emphasizes four core principles: transparency and explainability of algorithms, robust data privacy and security protections, equity and inclusiveness to counter algorithmic bias, and strong accountability mechanisms for AI developers and implementers [65]. These considerations are particularly critical in the context of NDDs, where patient data are highly sensitive and equitable access to diagnosis and treatment is essential.

Complementing the WHO guidance, the FUTURE-AI initiative—a global, multi-stakeholder consortium—developed international consensus guidelines to ensure the trustworthiness of AI in clinical practice. The framework outlines six core principles: fairness, universality, traceability, usability, robustness, and explainability, designed to guide the ethical and effective implementation of AI in healthcare, including in the management of NDDs [66].

In the United States, the Food and Drug Administration (FDA) acknowledged the growing prominence of AI in medical technologies through its AI/ML-Based Software as a Medical Device (SaMD) Action Plan. This plan outlines strategies to ensure good machine learning practices (GMLP) by establishing a tailored regulatory framework for AI/ML-based devices that fosters a patient-centered approach and promotes post-market performance monitoring [67]. These efforts are critical in ensuring that AI applications in NDD diagnostics and treatment are safe, effective, and continuously refined in real-world settings. The US White House reinforced these guidelines when it introduced the Blueprint for an AI Bill of Rights in October 2022. This policy framework articulates five fundamental principles: (1) Safe and effective systems, (2) Algorithmic discrimination protections, (3) Data privacy, (4) Notice and explanation, and (5) Human alternatives, consideration, and fallback. These principles provide a strong foundation for ensuring human dignity and fairness in AI applications across domains, including in the highly sensitive area of pediatric NDD care [68].

In Europe, the European Union’s proposed Artificial Intelligence Act represents one of the most comprehensive attempts to regulate AI technologies based on risk stratification [69]. Because AI systems are classified as “high-risk”, they will be subject to rigorous oversight, including requirements for high-quality, bias-mitigated training datasets, thorough documentation, transparency in decision-making processes, and mandated human oversight mechanisms [69]. The implications of this act are particularly relevant for developers and implementers of AI tools for NDD screening, diagnosis, and treatment.

Together, these regulatory and ethical frameworks offer a foundational structure for guiding the ethical and responsible development and deployment of GenAI in NDD care, ensuring that these technologies not only advance innovation but also uphold the highest standards of safety, equity, and accountability. However, organizations such as the WHO should take the lead in reviewing existing frameworks and developing region-specific guidelines that reflect local needs. Individual institutions must take responsibility for developing and operationalizing AI adoption policies. Institution-wide policies must be informed by both regional and global frameworks and should outline a phased adoption model with clearly defined timelines for technology acquisition, piloting, scale-up, evaluation, and full integration. Importantly, such frameworks must also address how institutions will manage current issues with accuracy, bias, hallucination, and data security associated with AI use.

## 5. Discussion

Integration of GenAI into the continuum of care for neurodevelopmental disorders (NDDs) holds transformative potential to improve nearly every facet of NDD management. To actualize this, stakeholders must invest in the development of tailored GenAI tools and resources designed specifically for children with NDDs, their care providers, and their families through strong, interdisciplinary collaboration between clinicians, AI experts, biomedical engineers, and behavioral scientists. Using an Inside-Out (clinicians and families) and Outside-In (industry) innovation approach, efforts should be geared towards co-design, co-creation, co-validation, stakeholder training, transformation, and appropriate deployment of AI models that are clinically relevant, ethically sound, and responsive to the diverse needs of neurodivergent populations [28,30]. Integrating GenAI into existing clinical workflows demands a reevaluation of current care pathways before embedding GenAI as a core function in pediatric neurodevelopmental services. Equally important is the establishment of comprehensive policy frameworks that mandate and regulate the ethical integration and use of GenAI in NDD care, ensuring safety, equity, and sustainability, especially in nations with established digital health infrastructure and AI capabilities. All supporting regulatory frameworks must mandate the safeguarding of data privacy, the need for informed consent from patients and guardians, and guarantee equitable access to AI-enhanced healthcare services [65,70].

Although there are significant risks and cost considerations associated with using AI in the management of NDDs, stakeholders are already making substantial investments in AI across several areas of healthcare. For example, in 2024, global venture funding for AI-related sectors exceeded $100 billion, making AI the top recipient of venture capital investment [71]. Failing to harness these resources for the development and deployment of AI in NDD care would represent a missed opportunity with serious consequences. To ensure equitable advancement, stakeholders must reallocate and prioritize funding to explicitly include NDD care as a key focus within broader AI development efforts.

Beyond regulations, AI systems that uphold algorithmic transparency, mitigate bias, and incorporate culturally sensitive, patient-centered design principles should be developed. Such safeguards are especially vital in NDDs where inappropriate or biased recommendations can have long-term developmental consequences [35,36]. A central ethical concern is ensuring that caregivers understand how AI technologies function, their limitations, and the implications of their outputs. Transparent communication and shared decision-making must remain foundational, particularly when AI-generated insights are used to guide sensitive clinical decisions involving children [72]. Moreover, ongoing empirical studies are needed to rigorously validate the safety, clinical effectiveness, and real-world impact of GenAI applications in neurodevelopmental care across diverse populations and settings [31,40]. Emerging regulatory standards and policy initiatives designed to advance AI in NDD care must strike a balance between innovation and responsibility, ensuring that AI benefits are distributed equitably and ethically among all children and families. However, stakeholders should be cognizant of data heterogeneity, scalability concerns, and interpretability of GenAI outputs, which may interfere with the adoption and assimilation of AI in NDD care.

To avoid potential unintended consequences of GenAI deployment in pediatrics, following the ISO 14971 guidelines is imperative. ISO 14971 offers structured direction on conducting risk-benefit analyses, selecting appropriate risk control measures, and utilizing production and post-production data for ongoing risk assessment [73]. This guideline forms the foundation for applying risk management principles throughout the entire lifecycle of medical devices, such as AI systems, from design and development to deployment and post-market monitoring. Developers and clinicians must adopt a systematic, evidence-based approach to identify AI-related risks such as bias, performance variability, hallucinations, and unintended consequences; evaluate their severity and likelihood; implement appropriate controls; and continually assess the effectiveness of those measures.

A product- and process-specific risk management framework is essential to meet safety and performance standards in alignment with global regulatory expectations. To ensure compliance and protect patient well-being, AI developers must embed this risk management process within their broader quality management systems. By taking these strategic steps, we can accelerate innovation in NDD management and bring measurable, lasting benefits to children and families navigating these lifelong conditions.

## 6. Conclusions

GenAI presents a transformative opportunity to address the complex and persistent challenges associated with NDDs in children. As an emerging technological frontier, GenAI has the potential to fundamentally reshape the landscape of pediatric neurodevelopmental care by advancing early detection, enhancing diagnostic precision, individualizing treatment protocols, accelerating inclusive research, and empowering families with accessible and responsive support tools. Ethical, equitable, and responsible GenAI adoption and integration can reduce disparities in care access, mitigate diagnostic delays, and improve therapeutic outcomes across diverse populations. Its application in real-time behavioral analysis, personalized intervention planning, and caregiver education offers a scalable and innovative solution to the global burden posed by NDDs.

The promise of GenAI lies not only in technological advancement but in its potential to deliver person-centered, culturally sensitive, and evidence-based care to millions of children and families worldwide. To realize this promise, it is imperative that multilateral organizations and governments of nations adopt a global implementation plan with a timeline that will see to the incorporation of AI in NDD by the end of 2027 across all nations. WHO and other regional bodies should establish monitoring dashboards to track AI adoption rates in NDD care, outcomes, and equity metrics. Also, urgent collaboration between clinicians, researchers, technologists, and policymakers towards integration of GenAI tools within existing care infrastructures while upholding the highest standards of safety, transparency, and inclusivity is needed. Moving forward, government, clinicians, and impacted families must embrace GenAI as a tool for progress. However, its deployment must be grounded in robust ethical frameworks and rigorous scientific validation. Doing so will ensure that the benefits of this innovation are not only transformative but also just and sustainable, contributing meaningfully to improved lifelong outcomes for individuals affected by NDDs.

As previously noted, there is a significant lack of reliable data on NDDs from sub-Saharan Africa and other LMICs. This data gap hinders the development of inclusive AI models and perpetuates existing biases within current algorithms. While over $100 billion was invested in 2024 in AI development, the vast majority of funding was concentrated in high-income countries (HICs), leaving LMICs underserved. To ensure equity and avoid widening already existing disparities, we must bridge this disparity through targeted investments designed to support research on NDDs and AI applications within sub-Saharan Africa. Furthermore, capacity-building initiatives must be implemented to train healthcare workers in LMICs on NDD screening, diagnosis, and management. In parallel, national, regional, and global organizations must prioritize the creation of a structured and timed roadmap for generating high-quality data and developing equitable AI models tailored to the realities of LMICs. Such efforts are critical to narrowing the current digital and AI divide between LMICs and HICs. Ultimately, leaders in LMICs should elevate NDD care and inclusive AI development as national priorities, recognizing them as essential components of sustainable development and self-determination.

## 7. Future Directions

Moving forward, there is an urgent need to develop a more diverse dataset to retrain NDD-specific GenAI Models. This will ensure appropriate data diversity, thus improving the generalizability of models and minimizing diagnostic disparities. A more diverse dataset will also ensure we design, develop, train, and deploy models that are inclusive and able to recognize neurodevelopmental variations across global and marginalized populations. In the mid-term, national and regional bodies should fund rigorous clinical trials and translational studies that evaluate the short-, medium-, and long-term effectiveness, safety, and clinical impact of GenAI applications in NDD care. Proposed studies should investigate not only diagnostic accuracy but also improvements in treatment adherence, developmental outcomes, caregiver well-being, health system efficiency, and potential unintended consequences of AI deployment and scale-up.

GenAI should be integrated into established multidisciplinary care teams and workflows as we explore how GenAI to facilitate interprofessional communication, streamline care coordination, and support shared decision-making across diverse care settings. Also, as consensus around the need for participatory approaches in AI development continues to emerge, initiatives should involve caregivers, educators, clinicians, and individuals with lived experience of NDDs in the design, testing, and oversight of GenAI tools. This co-design, co-development, co-validation, and transformative approach will improve ownership, relevance, trustworthiness, and cultural sensitivity. Also, the curricula for medical, allied health, and behavioral professionals should be updated to include training on the ethical, practical, and technical aspects of AI tools as we operationalize continuing education and certification programs to support safe and effective adoption.

Without appropriate inclusive policies, we may never achieve these noble objectives. We should leverage existing policy frameworks as well as modify them to support ethical GenAI integration. New policies must cover data governance and ownership, safety and privacy, ethical and responsible use of GenAI in NDD care, accuracy and hallucination, as well as reimbursement models for AI-enhanced care.

Finally, we should look beyond clinical care and use GenAI to support related public health initiatives in school systems, surveillance activities, community health programs, and digital health platforms. To achieve these, we must address the technical and infrastructural challenges of data interoperability as we work towards developing cohesive models that enhance predictive and personalized care.

## Abbreviations

The following abbreviations are used in this manuscript:

AI	Artificial Intelligence
ABA	Applied Behavior Analysis
ADDM	Autism and Developmental Disabilities Monitoring Network
ADHD	Attention-Deficit/Hyperactivity Disorder
ASD	Autism Spectrum Disorder
CDC	Centers for Disease Control and Prevention
EHR	Electronic Health Records
FDA	Food and Drug Administration
GenAI	Generative Artificial Intelligence
GMLP	Good Machine Learning Practices
ID	Intellectual Disability
NDDs	Neurodevelopmental Disorders
SLD	Specific Learning Disorders
SSA	Sub-Saharan Africa
WHO	World Health Organization
